# Augmented glycerosomes as a promising approach against fungal ear infection: Optimization and microbiological, *ex vivo* and *in vivo* assessments

**DOI:** 10.1016/j.ijpx.2024.100295

**Published:** 2024-10-22

**Authors:** Sadek Ahmed, Heba Attia, Osama Saher, Abdurrahman M. Fahmy

**Affiliations:** a Department of Pharmaceutics and Industrial Pharmacy, Faculty of Pharmacy, Cairo University, Egypt; b Department of Microbiology and Immunology, Faculty of Pharmacy, Cairo University, Cairo, Egypt; c Department of Laboratory Medicine, Karolinska Institute, Stockholm, Sweden; Department of Cellular Therapy and Allogeneic Stem Cell Transplantation (CAST), Karolinska University Hospital Huddinge and Karolinska Comprehensive Cancer Center, Stockholm, Sweden

**Keywords:** Voriconazole, Otomycosis, Factorial design, Transmission electron microscopy, Augmented glycerosomes, Minimal fungicidal concentration

## Abstract

In the current study, voriconazole (VCZ) augmented glycerosomes were optimized for topical otomycosis management according to a 2^3^ factorial design, employing a thin film hydration method. By optimizing Glycerol volume, limonene: VCZ ratio and Span® 60: soybean phosphatidyl choline (PC) ratio, glycerosomes with maximum percentage entrapment efficiency (%EE) and zeta potential (ZP) and minimum vesicle size (VS) and polydispersity index (PDI) were to be obtained. An optimal augmented glycerosomal formula (OAG) that contained 10 mg VCZ, 150 mg PC, and 3 mL glycerol, comprising 2.5: and 0.92:1 ratios of the latter two independent variables, was proposed via numerical optimization. OAG exhibited high %EE and ZP values and acceptable low values for VS and PDI (84.3 ± 2.0 %, −38.8 ± 1.8 mV, 191.0 ± 1.1 nm, and 0.192 ± 0.01, respectively). Extensive in *vitro* testing of OAG revealed the entrapment of VCZ within OAG, biphasic in *vitro* release profile, stability for up to 3 months at 2–8 °C and spherical morphology of OAG with VS like that obtained via zetasizer. OAG demonstrated higher permeated amounts of VCZ and flux values than VCZ suspension, leading to an enhancement ratio of 2.56 in the *ex vivo* permeation study. The deeper penetration ability of OAG demonstrated by Confocal Laser Scanning Microscopy and its superior in *vitro* antifungal activity confirmed the validity of the *ex vivo* study. Also, the histopathological study confirmed the safety of OAG for topical use, suggesting that VCZ OAG was a promising topical antimycotic formula.

## Graphical abstract

**Figure:**
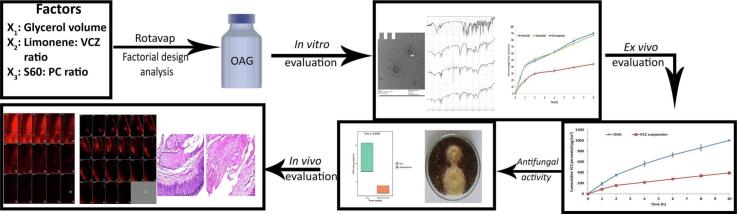

## 1 Introduction

*Candida* and *Aspergillus* spp. are the primary causative agents of fungal otitis externa (otomycosis), a condition commonly seen in primary care and otolaryngology clinics (Koltsidopoulos and Skoulakis, 2020). The widespread use of antibiotics and steroids following the COVID-19 pandemic has contributed to a continually growing number of cases, especially among immunocompromised individuals such as diabetics, leukemia and HIV patients, especially in regions with hot and humid climates (Aremu et al., 2020; Khasanov et al., 2022; Van Paassen et al., 2020). If left untreated or misdiagnosed, otomycosis can lead to severe complications, ranging from otic discharge, tinnitus, and tympanic membrane perforation to skull base invasion, nerve palsies, or even death (Parize et al., 2009). The usual intervention for otomycosis management involves initial local debridement combined with changing the pH and topical antifungal treatment. Acidifying the ear canal using acetic acid or other acidic solutions creates a less favorable environment for fungal growth. However, severe or refractory cases may require systemic administration of high doses of antifungals (Sander, 2001; Yassin et al., 2023). While older antifungals like amphotericin B have proven efficacy, they require the intravenous administration of large -usually toxic- doses, usually leading to renal failure, necessitating immediate withdrawal of therapy, or a significant dose reduction, frequently resulting in therapeutic failure (Parize et al., 2009; Sangaré et al., 2021). Triazole antifungals (especially voriconazole (VCZ)) have full activity against otic mycosis, including those caused by *Aspergillus* spp., and have an advantage over the commonly used imidazole type compounds (ketoconazole, clotrimazole, and econazole). VCZ's high lipophilicity is a key parameter in its skin permeation and fungal eradication, making it a drug of choice in such cases (*Log P* *=* 1.8) (Ali et al., 2018; Peyton et al., 2015; Veloso et al., 2018; Zhang et al., 2021). The absence of approved VCZ topical formulations drove otologists and ophthalmologists to use reconstituted VCZ solutions with cyclodextrins intended originally for intravenous administration or the use of 1 or 2 % suspension of VCZ in water for topical application. There are some reported off-labeled uses of VFEND® (a marketed Voriconazole powder for solution from Pfizer for infusion or oral suspension) for fungal ear infections (Drew and Perfect, 2022, Wee et al., 2023). These approaches need frequent application and high cost of therapy, eventually leading to patient non-compliance and therapeutic failure (Al-Badriyeh et al., 2009; de Sá et al., 2015; Neoh et al., 2016). Hence, it would be beneficial to utilize drug nanocarriers for the formulation of VCZ into suitable platforms to provide efficient delivery via different routes of administration. Previous works focused on utilizing nanocarriers for ophthalmic (Said et al., 2021), follicular (Santos et al., 2018), and intravenous delivery of VCZ (Esentürk et al., 2020; Veloso et al., 2018).

An attractive formulation described in recent years by multiple studies is glycerosomes (Manca et al., 2016; Melis et al., 2016; Naguib et al., 2020; Salem et al., 2018). They are formed by the incorporation of glycerol into liposomal formulations, which ensures a high degree of vesicular bilayer fluidity, leading to enhanced drug permeation through different biological membranes. (Zhang et al., 2017). Glycerol is known to have both immunomodulatory and anti-inflammatory actions. (Freitas et al., 2021; Widyarman et al., 2018). Also, through its cosolvent and viscosity-enhancing effects in formulations, it presents drugs at high concentrations at the site of action (Hao and Li, 2019).

To the best of our knowledge, no existing studies explore the use of voriconazole (VCZ) nanocarriers, particularly VCZ glycerosomes, as drug-delivery systems for otomycosis treatment. Therefore, our study aimed to investigate the potential of VCZ-augmented glycerosomes for topical otomycosis management. We enhanced glycerosomes by replacing cholesterol in traditional glycerosomes with Span® 60 (S60), a lipophilic non-ionic surfactant. This substitution maintains vesicular rigidity similar to cholesterol while producing vesicles with a high negative zeta potential and small size, which resist aggregation during storage (Yasser et al., 2024). Additionally, we incorporated limonene, a well-known lipophilic terpene permeation enhancer, to further improve VCZ permeation for more effective otomycosis treatment (Albash et al., 2021). We employed specialized statistical software (Design- Expert®) to define optimum levels of key formulation variables. A factorial design was preferred since it would optimize multiple variables simultaneously and measure any possible interactions between variables while minimizing the needed experimental runs to identify the optimized formula. This optimal formula would then be prepared and rigorously tested to ensure their stability, safety, and effectiveness (Krtalić et al., 2018). In summary, this study aims to address the limitations of current otomycosis treatments by developing a novel topical voriconazole (VCZ) formulation using glycerosomes, which enhance drug permeation and stability. The glycerosomes are optimized with Span® 60 for vesicular stability and limonene for improved VCZ penetration. This approach seeks to provide a more effective and patient-friendly alternative for treating fungal ear infections.

## 2 Materials and methods

### 2.1 Materials

Voriconazole (VCZ) was a kind gift from EVA Pharmaceuticals, Giza, Egypt. Soybean phosphatidyl choline (PC) (≥ 30 enzymatic), Span® 60 (S60), rhodamine B (RhB) and dialysis membrane (Mwt. Cutoff ≈ 12,000-14,000) were purchased from Sigma Aldrich, Saint Louis, Missouri, USA. Limonene was purchased from Loba Chemie, Mumbai, Maharashtra, India. Sabouraud Dextrose Broth (SDB) and Sabouraud Dextrose Agar (SDA) media were purchased from Oxoid, Hampshire, UK. Methanol, chloroform, glycerol, sodium dihydrogen phosphate, disodium hydrogen Phosphate, and sodium chloride were obtained from Al Nasr Chemical Company, Cairo, Egypt, and were of analytical grade.

### 2.2 Methods

#### 2.2.1 Experimental design

VCZ augmented glycerosomes (AG) were optimized via a 2^3^ full-factorial design using Design-Expert® 12 software (Stat-Ease, Inc., Minneapolis, MN), where glycerol volume, limonene: VCZ ratio and S60: PC ratio were the independent variables. Percentage entrapment efficiency (%EE), vesicle size (VS), polydispersity index (PDI) and zeta potential (ZP) were chosen to be the investigated responses. The variables, their codes and levels, together with the responses and their codes and desirability constraints, are listed in Table1. A 2^3^ factorial design was preferred due to both simplicity and effectiveness. The choice of the factors and levels to be included was based on a previously conducted preliminary study.

#### 2.2.2 Formulation of VCZ AG

VCZ AG were developed according to a typical thin film hydration method. The organic phase was composed of PC (150 mg), VCZ (10 mg), limonene, and S60 (Table 2), dissolved in 10 mL of a 7:3 chloroform and methanol mixture. Rotary evaporator (Rotavapor, Heidolph VV 2000, Burladingen, Germany) evaporation conditions were 60 °C, 120 rpm and 15 min. The aqueous phase (10 mL solution of glycerol in double-distilled water) was used as a hydration medium, employing the same conditions for 30 min without vacuum. (Bender et al., 2024). The developed dispersions were then refrigerated at 4 °C until further testing.

#### 2.2.3 Estimation of percentage entrapment efficiency

Indirect measurement of %EE of VCZ into AG was carried out. (El Menshawe et al., 2024). One mL sample of AG was ultra-centrifugated at 22,000 rpm at 4 °C for 2 h in a cooling ultra-centrifuge (3 K30, Sigma, Germany), followed by adequate dilution and spectrophotometric measurement of the unentrapped VCZ at 276 nm (λ_max_ of VCZ). The %EE of VCZ was obtained from the following equation:

(1) $$\%\mathrm{EE}=\left(\;\left(\mathrm{Total drug content}-un\mathrm{entrapped amount}\right)/\left(\mathrm{Total drug content}\right)\right)\times 100$$

#### 2.2.4 Measurement of vesicle size, polydispersity index, and zeta potential

Zetasizer (Model ZEN3600, Malvern Instruments Ltd. Worcestershire, UK) was used to determine VS and PDI for 100× double-distilled water dilutions of AG formulae. The same equipment utilizing a special cuvette was used for ZP measurements (Ahmed et al., 2024, El Hosary et al., 2024). All experiments were carried out in triplicates.

#### 2.2.5 Statistical optimization of VCZ AG

The design constraints in Table 1 were fed to the numerical optimization function in Design-Expert® software to suggest an optimal VCZ augmented glycerosomes formulation (OAG), whose observed and predicted responses were then correlated via calculating the deviation percentage using the following equation:

(2) $$\mathrm{Deviation percentage}=\left(|\mathrm{Predicted value}-\mathrm{Observed value}|/\left(\mathrm{Observed value}\right)\right)\times 100$$

**Table 1:** Factorial design used for optimization of voriconazole augmented glycerosomes.

Factors (independent variables)	Levels	
	(−1)	(+1)
X_1_: Glycerol volume (mL)	1.5	3
X_2_: Limonene: VCZ ratio	2.5	5
X_3_: S60: PC ratio	0.5	1


Responses (dependent variables)	Desirability Constraints
Y_1_: Percentage entrapment efficiency (%EE) (%)	Maximize
Y_2_: Vesicle size (VS) (nm)	Minimize
Y_3_: Polydispersity index (PDI)	Minimize
Y_4_: Zeta potential (ZP) (mV)	> 35 (absolute value)

VCZ: voriconazole, S60: Span® 60, PC: Phosphatidyl choline.

Afterwards, OAG was subjected to extensive in *vitro*, *ex vivo* and *in vivo* testing.

#### 2.2.6 Physicochemical properties of VCZ-loaded OAG

The morphology and VS of VCZ OAG were observed using a transmission electron microscope (JEOL, Tokyo, Japan) operating at 80 kV after adequate dilution, deposition on a carbon-coated copper grid, and staining with 2 % *w*/*v* phosphotungestic acid. (Yousry et al., 2023, Younes et al., 2024).

Fourier transform infrared spectroscopy (FTIR) peaks of VCZ, S60, PC, and lyophilized OAG were detected by FTIR spectrophotometer (model 22, Bruker, Coventry, UK), to detect possible interactions between VCZ and the used components and to demonstrate the VCZ entrapment into OAG. Detections were carried out at 25 °C in the range of 4000–500 cm^−1^ after adequate desiccation and compression of samples into KBr discs. (Ahmed et al., 2024).

The pH value of OAG was measured using a Jenway® 3510 benchtop pH meter (Cole-Parmer®, Illinois, USA) to ensure its compatibility with the skin, causing no irritation. (Fouda et al., 2018).

The in *vitro* release of VCZ from OAG was compared to its release from an aqueous suspension of the same concentration and viscosity (1 *mg/mL* and 1.53 *cp*, respectively) prepared by dispersing VCZ into 0.1 % *w/w* methylcellulose (MC) aqueous dispersion, using dialysis bag method. Dialysis bags (Mwt. Cutoff ≈ 12,000-14,000) of 5 cm length served as a donor compartment, housing 1.5 mL OAG or VCZ suspension (equivalent to 1.5 mg VCZ) and were immersed in the receptor compartment (50 mL phosphate-buffered saline (PBS) (pH = 7.4) contained in amber-colored bottles). These were then mounted in a shaking water bath (37 °C and 50 strokes per minute and sample volume of 3 mL at 0.5, 1, 2, 4, 6, and 8 h), followed by spectrophotometric determination at 276 nm. To maintain sink conditions, fresh PBS was added after each sample was taken. (Ahmed and Badr-Eldin, 2019). After that, plotting % VCZ released vs. time (h) was done to obtain release profiles, where the best fitting release model (zero order, first order, or Higuchi diffusion) would be that of the largest coefficient of determination (R^2^) (Ahmed et al., 2020).

The experimental setup to check the stability of our OAG followed previous protocols reported in literature for glycerosomes (Manca et al., 2013; Naguib et al., 2021) or VCZ in other formulations (de Sá et al., 2015; Khare et al., 2016). We refrigerated OAG (2–8 °C) for 3 months, after which visual inspection for macromorphological changes (color change and aggregate formation) was carried out to conclude the preservation of physical characteristics (Albash et al., 2022). Student's *t*-test, setting α at 0.5, was carried out to compare %EE, VS, and PDI of fresh and stored OAG. Finally, the following equation was utilized to obtain the similarity factor (*f*_2_) between the in *vitro* release profiles of fresh and stored OAG (Abdelbary et al., 2016):

(3) $$f_{2}=50.\log\{{\left[1+\left(1/n\right)\sum_{t=1}^{n}{\left(R_{t}-T_{t}\right)}^{2}\right]}^{-0.5}.100$$

#### 2.2.7 VCZ *ex vivo* permeation through hairless rabbit ear skin

Albino rabbit ear skin from six freshly slaughtered rabbits at a local slaughterhouse (animals intended for consumption as food) was dissected, cleaned, and divided into pieces of appropriate sizes after hair removal and visual check under a light microscope for blemishes. The pieces were mounted on smooth-edged plastic tubes of 0.5 cm radius, with the stratum corneum facing upwards, inside which 1.5 mL of OAG or VCZ aqueous suspension (equivalent to 1.5 mg VCZ) was added, acting as a donor compartment. The donor compartment was then partially submerged in the receptor compartment (a glass beaker containing 25 mL of PBS of pH 7.4. A magnetic stirrer operating at 37 °C and 50 rpm was used, with 1, 2, 4, 6, 8, and 10 h sampling intervals, each of 2 mL. Samples were replaced with fresh PBS for maintenance of sink condition. After that, the samples were processed by mixing with equal volumes of methanol and sonication for 3 min for coagulating any leached skin proteins, followed by centrifugation (15 min at 4000 rpm). Supernatants were spectrophotometrically analyzed at 276 nm. Finally, the cumulative amounts of permeated VCZ per surface area (μg/cm^2^) vs. time (h) were plotted to obtain permeation profiles, followed by employing the following equations to conclude the maximum flux at 10 h (*J*_max_) and the enhancement ratio (ER) (Al-Mahallawi et al., 2021):

(4) $$J_{\max}=\left(\mathrm{Cummulative}\;\mathrm{VCZ}\;\mathrm{amount permeated}\;\mathrm{at}\;10h\right)/\left(\mathrm{final time}\times \mathrm{permeation area}\right)$$

(5) $$\mathrm{ER}=\left(J_{\max}\;\mathrm{OAG}\right)/\left(J_{\max}\;\mathrm{VCZ}\;\mathrm{suspension}\right)$$

#### 2.2.8 In *vitro* antifungal activity study of VCZ OAG

##### 2.2.8.1 Fungal Strain and inoculation conditions

In this study, the tested fungal strain was *Aspergillus niger* standard strain (ATCC32656), and the fungal growth media were Sabouraud Dextrose Agar (SDA) and Sabouraud Dextrose Broth (SDB). All inoculated growth media were incubated for 48–96 h at 28 ± 2 °C.

##### 2.2.8.2 Determination of the antifungal activity using Kirby–Bauer disk diffusion susceptibility test

The standard Kirby–Bauer disk diffusion test was used to evaluate the effect of OAG on the antifungal activity of VCZ, according to the Clinical and Laboratory Standards Institute guidelines. (CLSI, 2017). Briefly, the fungal spore turbidity in Sabouraud Dextrose Broth (SDB) was adjusted to 0.5 McFarland by measuring and adjusting the absorbance to be in the range of 0.1–0.125 at wavelength 550 nm. Sabouraud Dextrose Agar (SDA) plates were then surface-inoculated with the turbidity-adjusted fungal culture. Sterile filter paper discs were loaded with 10 μL of either OAG or VCZ suspension and then allowed to dry for 10 min. The dried discs were then dispensed on the fungal-inoculated SDA plates. The plates were incubated at 28 ± 2 °C for 48–96 h, after which the diameters of the zone of inhibition were measured and recorded. The experiment was carried out in triplicate. Blank control with OAG excipients was conducted.

##### 2.2.8.3 Determination of the Minimum Inhibitory Concentration (MIC)

Microbroth dilution technique was adopted to determine minimum inhibitory concentrations of both VCZ OAG and VCZ suspension, according to the guidelines described by the Clinical and Laboratory Standards Institute (CLSI, 2017). In U-shaped bottom, sterile 96-well plates, two-fold serial dilutions for both OAG and VCZ suspension (ranging from 500 to 0.28 μg/mL) in 100 μL of double-strength Sabouraud Dextrose Broth (SDB) were prepared. For the inoculum preparation, several discrete spores from the surface of an inoculated Sabouraud Dextrose Agar plate were cultured in SDB and incubated for 48 h at 28 ± 2 °C. The turbidity of the fungal suspension was then adjusted with SDB to obtain an optical density of 0.08–0.13 at wavelength of 600 nm; then, the turbidity-adjusted culture was further diluted 1:100 to bring a 10^5^–10^6^ 6 CFU/mL inoculum size. Ten microliters of the 100 times diluted fungal spores were dispensed to each well of the 96-well microtitre plates. A control for the viability of the fungal culture (positive control), a control for the sterility of the used growth media (negative control), and a blank control with OAG excipients were conducted. Then, the microtitre plates were incubated at 28 ± 2 °C for 48 h, after which each well's turbidity was recorded, and the absorbance was measured at a wavelength of 600 nm using an automated 96-well plate reader. Tests were performed in triplicates (biological and technical replicas). The MIC values for each preparation against *Aspergillus niger* were determined by calculating the lowest concentration at which the tested fungal culture demonstrated no visible growth. Statistical analyses were performed in version 4.1.2 of R (R Core Team, 2022) and visualized in RStudio (Racine, 2012; Rstudio, 2022).

##### 2.2.8.4 Minimum Fungicidal Concentration (MFC)

For both formulae (OAG and VCZ suspension), the Minimal Fungicidal Concentration (MFC) was determined by broth microdilution method in accordance with the guidelines set out by the Clinical and Laboratory Standards Institute (CLSI, 2017). Ten microliters of 10^5^–10^6^ CFU/mL fungal spore culture was incubated in a sterile 96-well plates with twofold dilutions of both the drug and optimized formula. The plates were then incubated at 28 ± 2 °C for 48 h, after which a 10 μL from each well was spotted on SDA plates and incubated again at the same incubation conditions for 48 h. Biological and technical triplicates of the experiment were conducted. The lowest concentration that showed no fungal growth on the SDA plates was recorded as MFC.

##### 2.2.8.5 Time to kill assay

The time needed for both OAG and VCZ suspension to kill the inoculated fungal culture was determined according to the method described by Osman et al., (Osman et al., 2023) with some modifications. Briefly, two 1500 μL SDB Eppendorf tubes containing a concentration equals to the MIC of both tested preparations, and a third tube serving as a positive control (contain neither), were separately inoculated with 150 μL of 10^6^ CFU/mL fungal spore culture. Ten microliters from each preparation mixture were ten-fold diluted and spotted on SDA agar plate (zero-time sample). The Eppendorf tubes were then incubated at 28 ± 2 °C and 10 μL from each tube were withdrawn at time intervals of 4, 6, 10, 16, 24, 48, 30, 36 and 72 h after the incubation. The withdrawn sample of each time interval were first diluted 10 times in sterile saline solution, and then 10 μL of the tenfold-diluted solution were spotted on SDA agar plate. The experiment was conducted in triplicate. The observed fungal growth for each time sample was recorded.

#### 2.2.9 *In vivo* safety and performance studies of VCZ OAG

##### 2.2.9.1 Animals

The protocol for the *in vivo* study was approved by Research Ethics Committee of Faculty of Pharmacy, Cairo University (REC-FOPCU), Egypt (PI 3503), in compliance with ARRIVE guidelines and were carried out in accordance with the U.K. Animals (Scientific Procedures) Act, 1986 and associated guidelines, EU Directive 2010/63/EU for animal experiments.

Six male albino rabbits weighing 2–2.5 Kg were individually placed in wire cages with standard temperature, humidity, lighting and with food and water provided ad libitum. The ear skin was carefully shaved with electric clippers for hair removal followed by visual inspection for cuts and blemishes using a slit lamp. The animals were subjected to general anesthesia (xylazine (5 mg/kg) and ketamine (35 mg/kg)), prior to sacrifice (Albash et al., 2021).

##### 2.2.9.2 Histopathological examination

Pharmaceutical preparations- in addition to their effectiveness- should be of appropriate safety. For this purpose, OAG was tested for causing histopathological alterations/ damage to rabbit ear skin. In brief, two drops (100 μL) of OAG were instilled in the shaved left ears of three rabbits thrice per day for the period of a week while normal saline (negative control) was instilled in the right ears in the same manner. At the end of the experiment, the animals were anesthetized and euthanized as mentioned above. Then autopsy samples were taken from ear skin, fixed in 10 % formol saline, and then dehydrated using serial dilutions of ethanol. These were embedded in paraffin wax blocks, sliced using a microtome, followed by staining using hematoxylin and eosin and examination under light electric microscope (Abdelbary and AbouGhaly, 2015).

##### 2.2.9.3 *In vivo* skin penetration

Deep permeation of ototopical formulations is a reliable indicator of the formulation ability to deliver the target medicament to deep ear skin layers and, hence ensuring effective therapy. A confocal laser scanning microscope (CLSM) (LSM 710; Carl Zeiss, Jena, Germany), employing helium–neon (595 nm) and argon lasers (485 nm) was exploited for the estimation of permeation depth of 0.1 % *w/w* of Rhodamine B (RhB) in water (control), or replacing VCZ in OAG. Briefly, the right ear of each of three rabbits received a single, two drop (100 μL) dose of RhB solution, while the left ear received the same dose of OAG containing RhB instead of VCZ. After 3 h, the animals were anesthetized and euthanized. The ear skin was removed, washed with 10 % ethanol, gently dried then visualized (Sayed et al., 2021).

## 3 Results and discussion

### 3.1 Formulation of VCZ augmented glycerosomes

VCZ augmented glycerosomes were successfully prepared using thin film hydration method. The S60 and PC content in formulations resulted in off-white (yellowish) dispersions. The 2^3^ factorial design yielded eight formulations (coded AG1-AG8). Table 2 contains the compositions and measured responses for the eight experimental runs. Due to its indication of relative homogeneity in all formulations with values ranging from 0.221 ± 0.00 to 0.423 ± 0.06, PDI was excluded from the optimization process (Alyami et al., 2024).

**Table 2:** Experimental runs, independent variables, and measured responses of voriconazole augmented glycerosomes.

Formula	Composition			Y_1_: %EE	Y_2_: VS (nm)	Y_3_: PDI	Y_4_: ZP (mV)
	X_1_: Glycerol volume (mL)	X_2_: Limonene: VCZ ratio	X_3_: S60: PC ratio				
AG1	1.5	2.5	0.5	89.1 ± 1.5	224.9 ± 1.1	0.221 ± 0.00	−28.0 ± 0.1
AG2	1.5	5	0.5	81.6 ± 2.1	221.0 ± 4.3	0.227 ± 0.00	−34.9 ± 0.5
AG3	3.0	2.5	0.5	87.1 ± 1.8	200.0 ± 0.7	0.252 ± 0.02	−31.9 ± 0.6
AG4	3.0	5	0.5	77.1 ± 2.4	199.1 ± 0.8	0.242 ± 0.01	−36.7 ± 0.4
AG5	1.5	2.5	1	92.5 ± 2.8	218.0 ± 0.5	0.293 ± 0.01	−36.7 ± 1.6
AG6	1.5	5	1	79.5 ± 3.2	204.7 ± 2.2	0.234 ± 0.01	−48.6 ± 2.1
AG7	3.0	2.5	1	84.3 ± 2.0	191.0 ± 1.1	0.194 ± 0.00	−38.8 ± 1.8
AG8	3.0	5	1	76.0 ± 1.6	190.0 ± 8.3	0.423 ± 0.06	−56.6 ± 1.2

Presented values are the mean ± SD (*n* = 3).

VCZ: Voriconazole, S60: Span® 60, PC: Phosphatidyl choline, %EE: percentage entrapment efficiency, VS; vesicle size, PDI: polydispersity index, ZP: zeta potential.

### 3.2 Factorial design interpretation

The Design-Expert® software suggested the main effect model for the analysis of all responses. The design output data in Table 3 revealed the ability of the model to navigate the design space, with good ability to predict the values of the remaining responses (Y_1_: %EE, Y_2_: VS and Y_4_: ZP). These were concluded by model F-values (larger than 4) and proximity between predicted and adjusted R^2^ values (difference less than 0.2).

**Table 3:** Output data of the factorial design analysis of voriconazole augmented glycerosomes.

Response	Y_1_: %EE	Y_2_: VS (nm)	Y4: ZP (absolute value) (mV)
R-squared	0.9504	0.9604	0.9159
Adjusted R-squared	0.9132	0.9306	0.8528
Predicted R-squared	0.8015	0.8414	0.6635
Adequate Precision	12.2	14.8	10.5
Significant factors	X_1_, X_2_	X_1_, X_3_	X_2,_ X_3_
Regression equation (in terms of coded factors)	%EE = +83.41 −2.28 * A −4.85 * B −0.35 * C	VS = +205.99 −11.09 * A −2.44 * B −5.23 * C	ZP = +39.12 +1.87 * A +5.06 * B +6.27 * C

%EE: percentage entrapment efficiency, VS: vesicle size, ZP: zeta potential, A = X_1_: Glycerol volume, B = X_2_: Limonene: Voriconazole ratio, C = X_3_: Span® 60: Phosphatidyl choline ratio.

#### 3.2.1 %EE model interpretation

**Fig. 1:**
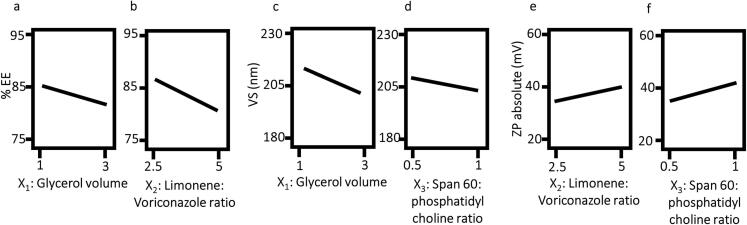
The effect of different significant formulation variables on: (a and b): percentage entrapment efficiency, (c and d): vesicle size and (e and f): zeta potential of voriconazole augmented glycerosomes.

The %EE of VCZ AG formulations ranged from 76.0 ± 1.6 to 92.5 ± 2.8 % (Table 2). The output data in Table 3 and Fig. 1a and b show that both X_1_: glycerol volume and X_2_: limonene: VCZ ratio had a significant effect on %EE of AG (*p* = 0.0206 and 0.0014, respectively). On the other hand, X_3_: S60: PC ratio had no significant effect on %EE, indicating that both components are efficient in entrapping VCZ (*p* = 0.5987).

The inclusion of limonene (terpene) in formulations is expected to promote a fluidized, lipophilic environment, due to its lipophilic nature (log *p* = 4.57). Such an environment provides suitable orientation for the C_16_ atom of the PC acyl chain, enhancing the entrapment of VCZ. However, increasing the limonene proportion would lead to destabilization and pore formation in the lipid bilayers of AG, eventually lowering %EE (Ahmed et al., 2023; Albash et al., 2021; Dragicevic-Curic et al., 2011).

These findings are in agreement with Albash et al., and Rangismawong et al., who found that increasing terpenes proportions lead to decreased %EE of fenticonazole nitrate and fluorescein sodium in their terpesomes and PEGylated liposomes, respectively (Albash et al., 2021; Rangsimawong et al., 2014).

Increasing glycerol amount would make it more and more available in the intervesicular medium leading to dissolution of greater amounts of VCZ, decreasing its %EE, due to its cosolvent properties (Manca et al., 2014; Naguib et al., 2020).

Similar results were achieved by multiple studies, where increasing glycerol amounts led to lowering of drug %EE within the corresponding glycerosomes (Moolakkadath et al., 2020; Salem et al., 2018; Younes and Habib, 2022).

Also, it's worth noting that it's common to encounter the opposite situation, where increasing glycerol amounts would increase %EE (Abdellatif et al., 2017; Manca et al., 2016). This leads to the conclusion that glycerol amounts should be carefully optimized in formulations. The %EE of our optimized glycosomes was comparable efficiency to other reported glycosome formulations (Salem et al., 2022; Zaki et al., 2022), voriconazole-loaded lipid nanoparticles (Khare et al., 2016).

#### 3.2.2 VS model interpretation

VS ranged from 189.6 ± 8.3 to 224.9 ± 1.1 nm (Table 2). The output data in Table 3 and Fig. 1c and d show that both X_1_: glycerol volume and X_3_: S60: PC ratio had a significant effect on VS of AG (*p* = 0.0009 and 0.0147, respectively). On the other hand, X_2_: limonene: VCZ ratio had no significant effect on VS (*p* = 0.1273).

The inverse relation between glycerol volume and VS can be attributed to the ability of glycerol to decrease the vesicular membrane rigidity, leading to the formation of smaller vesicles (Abdellatif et al., 2017).

The same finding was obtained by Younes and Habib, who found that increasing glycerol concentration led to the formation of smaller sertaconazole nitrate glycerosomes (Younes and Habib, 2022).

Again, it's widely noted that opposite results were obtained in multiple studies, where there was a direct relation between glycerol amounts in formulations and VS, confirming the importance of the optimization of glycerol amounts in formulations (Manconi et al., 2018; Moolakkadath et al., 2020; Zhang et al., 2017).

Increasing S60: PC ratio yielded vesicles of significantly smaller sizes. S60 is a hydrophobic surfactant (HLB = 4.7), such a low value decreases surface-free energy on the vesicular membranes, leading to smaller VS of AG. This effect is augmented by the partial negative charge on the polar heads of S60, preventing the formation of vesicular aggregates (Abdelrahman et al., 2017; Yoshioka et al., 1994).

Similar results were observed by Khazaeli and Pradakhty, and Ruckmani et al., who found that decreasing the HLB value of surfactants yielded smaller VS of caffeine and cytarabine hydrochloride niosomes, respectively (Khazaeli et al., 2007; Ruckmani et al., 2000).

#### 3.2.3 ZP model interpretation

High absolute ZP values are usually required to prevent vesicular aggregation in colloidal dispersions (Dubey et al., 2018). In the present study, ZP of formulations ranged from −28.0 ± 0.1 to −56.6 ± 1.2 mV (Table 2), which agreed with the mentioned requirement. The output data in Table 3 and Fig. 1e and f show that both X_2_: limonene: VCZ ratio and X_3_: S60: PC ratio had a significant effect on ZP of AG (*p* = 0.0157 and 0.0075, respectively). On the other hand, X1: glycerol volume had no significant effect on ZP of AG (*p* = 0.2101).

Increasing limonene ratio in formulations yielded more negative ZP values. Sakdiset et al. found that increasing the percentage of limonene from 5 to 10 % increased the negativity of ZP of lidocaine hydrochloride proniosomal gels (Sakdiset et al., 2023). Similar results were obtained by Prasanthi et al., upon increasing the limonene concentration from 0.5 to 1.5 % during the preparation of finasteride invasomes for iontophoretic transdermal delivery (Prasanthi and Lakshmi, 2013).

Increasing S60: PC ratio led to higher negative ZP values. The partially negative groups on the polar heads of S60 direct themselves toward the aqueous medium, imparting negative surface charges to the vesicles (Abdelrahman et al., 2017). Similar results were obtained by Magdy et al. upon the preparation of triamcinolone acetonide glycerospanlastics (Magdy et al., 2023).

### 3.3 Elucidation of optimal VCZ augmented glycerosomes formulation via numerical optimization

The numerical optimization process, based on the desirability criterion, was employed by Design-Expert® software to elucidate an optimal VCZ augmented glycerosomal formulation (OAG), according to the mentioned constraints in Table 1. The software suggested a formula that had 3 mL of glycerol, 2.5:1 ratio of limonene to VCZ and 0.92:1 ratio of S60 to PC, which had the highest desirability value of 0.805, The deviation percentage results in Table 4 demonstrated the closeness between the predicted and observed responses, concluding the validity of the elucidated models.

**Table 4:** The predicted and the observed responses of the optimal voriconazole augmented glycerosomal formula (OAG).

Response*	%EE	VS (nm)	ZP (mV)
OAG Observed value	84.3	191.0	−38.8
OAG Predicted value	85.6	192.1	−40.0
Percentage deviation	1.56	0.58	3.0

%EE: percentage entrapment efficiency, VS: vesicle size, ZP: zeta potential.

### 3.4 Physicochemical properties of VCZ-loaded OAG

**Fig. 2:**
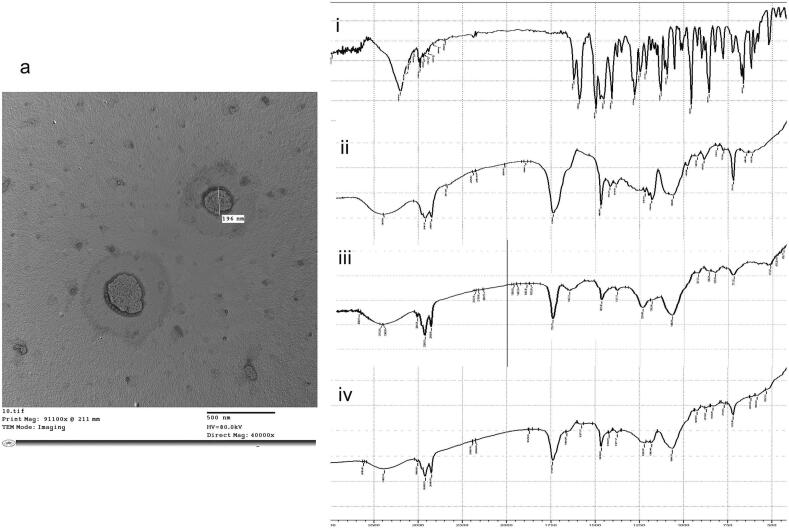
(a) Transmission Electron Microscope micrograph of optimal voriconazole augmented glycerosomes and (b) Fourier Transform Infrared spectra of i: pure voriconazole, ii: Span® 60, iii: soybean phosphatidyl choline and iv: lyophilized optimal voriconazole augmented glycerosomes.

The TEM image of OAG (Fig. 2a) demonstrated the presence of almost spherical vesicles, that had sizes similar to those obtained from zetasizer (around 200 nm), with hazy diffusional layers around them confirming both the validity of VS measurement and the sustained in *vitro* release pattern. Also, the absence of vesicular aggregates under the microscope reflected the highly negative ZP of OAG (−38.8 ± 1.8 mV) (Albash et al., 2022; Sakr et al., 2023).

The FTIR spectrum of VCZ (Fig. 2b(i)) demonstrates distinctive peaks at 3201.83 cm^−1^ (O—H stretching), 1495.76 to 1589.34 cm^−1^ (C=C stretching), 1276.88 to 1246.02 cm^−1^ (aryl C—N stretching), and at 1408.04 to 1130.29 cm^−1^ (C—F stretching) (Sun et al., 2016). The characteristic peaks of Span 60 (Fig. 2b(ii)) are observed at 3310 cm^−1^ (amines or hydroxyl group), 2926 cm^−1^ (carboxylic acids), 2360 cm^−1^ (primary and secondary amines), and 1733 cm^−1^ (aldehyde). The characteristic peaks of PC (Fig. 2b(iii)) are observed at 1069 cm^−1^ (C—O), 1465 cm^−1^ (C=C), 1740 cm^−1^ (C=O), and 2924 cm^−1^ (C—H). The characteristic peaks of VCZ were diminished in OAG spectrum (Fig. 2b (iv)), compared to pure VCZ, concluding the dispersion and entrapment of VCZ within OAG vesicles (Kaur et al., 2022; Sharma et al., 2023).

The pH value of OAG was measured to be 5.77 ± 0.08, which was suitable for topical otic applications.

To obtain optimum release profiles, the inclusion of both hydrophilic components that facilitate an initial burst release of the drug and lipophilic ones that secure a sustained release of the remainder of the dose should be carefully considered, and by keeping such a fine balance, efficient drug therapy can be achieved (Al-Mahallawi et al., 2017; Ammar et al., 2020; Tawfik et al., 2018).

**Fig. 3:**
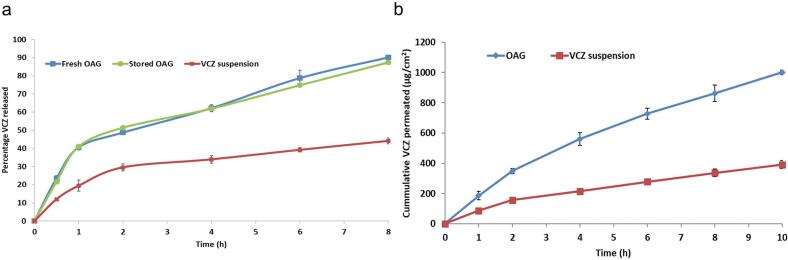
(a) In *vitro* release profiles of fresh and stored optimal voriconazole augmented glycerosomes, compared to voriconazole suspension and (b) *Ex vivo* permeation profiles of optimal voriconazole augmented glycerosomes and voriconazole suspension.

Upon the comparison of the in *vitro* release profiles of VCZ from fresh OAG and VCZ suspension (Fig. 3a), it can be seen that the poor aqueous solubility of VCZ led to failure of VCZ suspension to release adequate amounts of the drug over the period of the study (44.1 ± 1.6 % after 8 h). On the contrary, OAG released higher amounts of VCZ at all time intervals, with two distinguishable phases noticed. The first phase (initial burst) lasted for the first 2 h, with 48.8 ± 0.8 % VCZ released. Factors arising from the use of the hydrophilic component, glycerol, were usually behind this burst phase. These include the release of the fractions of dissolved unentrapped drug in the intervesicular medium, drug that was externally adsorbed on glycerosomal surfaces and drug entrapped between the vesicles' bilayers (ElKasabgy et al., 2014). The second phase (sustained release) for up to 8 h with 90.2 ± 0.6 % VCZ was then noted. Factors arising from the use of lipophilic components (limonene, PC and S60) were responsible for such sustainment. These include the combined effect of the release of VCZ from within the rigid, hydrophobic cores of the vesicles' bilayers and its hindered diffusion across the semipermeable membrane of dialysis bag, owing to the elevated medium viscosity (Ammar et al., 2020; ElKasabgy et al., 2014).

Similar results were obtained by Naguib et al., Albash et al., and Gupta et al., upon the fabrication of lacidipine glycerosomes, fenticonazole nitrate terpesomes and fluconazole niosomes, respectively (Albash et al., 2021; Gupta et al., 2011; Naguib et al., 2020).

It's worth noting that Higuchian diffusion was the best fitting release model for both OAG and VCZ suspension (had the largest R^2^ values).

For up to 3 months, OAG maintained its appearance and color. It was easily re-dispersed without any visible aggregates, owing to its highly negative ZP. Table 5 Compares the %EE, VS and ZP values for fresh and stored OAG. There was no significant change (*p* > 0.05) in the mentioned values at α = 0.05. Also, fresh and stored OAG had similar in *vitro* release profiles (Fig. 3a), indicated by a similarity factor of 79.2 (more than 50) (ElKasabgy et al., 2014). The above results conclude the stability of OAG (Harisa and Badran, 2015; Sayed et al., 2020). Additionally, there were no significant differences in the parameters of the reconstituted formula and the fresh one, implying the stability of lyophilized formulae (Supplementary File 1).

**Table 5:** Effect of storage on different measurements of voriconazole optimal augmented glycerosomes.

Parameter⁎	Fresh OAG	Stored OAG (3 months in refrigerator)	*p*- value
Percentage entrapment efficiency	84.3 ± 2.0	81.8 ± 1.2	0.267
Vesicle size (nm)	191.0 ± 1.2	194.9 ± 5.2	0.411
Zeta potential (mV)	−38.8 ± 1.9	−34.9 ± 0.5	0.099

OAG: Optimal augmented glycerosomes.

⁎ Mean ± SD (*n* = 3).

### 3.5 VCZ *ex vivo* permeation through hairless rabbit ear skin

Fig. 3b compares the *ex vivo* permeation profiles of VCZ from OAG and its suspension. It's evident that OAG allowed for the permeation of higher amounts of VCZ at all time intervals, confirming the results of the in *vitro* release study. OAG had shown significantly higher values of total cumulative VCZ permeated per surface area and maximum flux (*J*_max_) (1001.6 ± 14.2 μg/cm^2^ and 100.2 ± 1.4 μg/cm^2^/h, respectively), compared to VCZ suspension (391.3 ± 25.8 μg/cm^2^ and 39.1 ± 2.6 μg/cm^2^/h, respectively), leading to an enhancement ratio (ER) of 2.56.

The augmented glycerosomal vesicles, compared to VCZ suspension, exhibited smaller VS and hence a larger surface area, allowing for a deeper deposition of larger amounts of VCZ into skin layers (Agnihotri and Vavia, 2009).

By the inclusion of glycerol, limonene and S60, the augmented glycerosomes in this study combined the advantages of glycerosomes, terpesomes and spanlastics as well, leading to an efficient *ex vivo* permeation profile. These formulation components played a marked role in delivering VCZ in considerable amounts to the rabbit's ear skin. Due to its viscous and hygroscopic nature, glycerol boosted the fluidity and flexibility of glycerosomes' bilayers, together with their affinity to deeper cutaneous water-based layers, eventually aiding the vesicles to squeeze themselves through skin layers (Manca et al., 2013; Zhang et al., 2017). In addition, the incorporation of limonene, a renowned terpene permeation enhancer, acts by interruption of the hydrogen bonds of the skin lipid bilayers, thus disrupting the high order of the stratum corneum, and increasing the diffusivity of VCZ (Ahmed and Badr-Eldin, 2019; Ibrahim et al., 2024). Also, the incorporation of S60 yielded smaller vesicles and added to the vesicles' elasticity and their efficient squeezing through skin layers (Kaur et al., 2012). Moreover, small vesicles lead to deeper drug skin penetration via their rapid disintegration on the skin surface allowing for efficient material exchange between the vesicles and the intercellular lipidic components of the stratum corneum, and the subsequent introduction of drug molecules to deep skin layers (Manconi et al., 2006). Finally, the glycerol content aided the latter effect via the amalgamation of S60 (Ansari et al., 2022). The above results suggest a less frequent application of VCZ OAG, compared to VCZ suspension, eventually leading to patient compliance and successful therapy.

### 3.6 In *vitro* antifungal activity study of VCZ OAG

#### 3.6.1 Determination of the antifungal activity using Kirby–Bauer disk diffusion susceptibility test

**Fig. 4:**
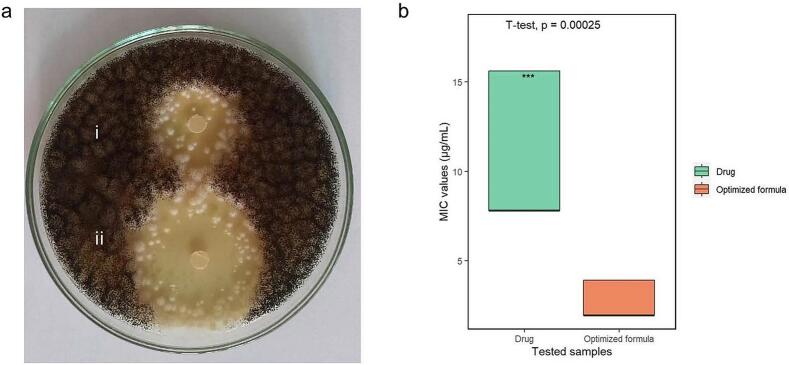
(a) Antifungal activity determination by Kirby–Bauer disk diffusion technique showing inhibition zones upon the loading of 5 μg of i: voriconazole suspension and ii: optimal voriconazole augmented glycerosomes on sterile filter paper disks. (b) Minimal inhibitory concentration values (μg/mL) of both the voriconazole suspension and optimal voriconazole augmented glycerosomes (Statistical significance assessed by independent *t*-test (*n* = 9), *p* = 0.00025).

The measured zone of inhibition of OAG (31.6 ± 0.24 mm) was significantly bigger than that of VCZ suspension (20.3 ± 0.26) (*t*-test, *p* value<0.05, Fig. 4a). This finding indicated an enhancement of the antifungal activity with OAG and confirmed the validity of the *ex vivo* permeation study. PC is lipidic in nature. Also, S60 and limonene are lipophilic compounds. All of these allow the vesicles to deeply interact with fungal cell walls and membranes, enhancing VCZ fungal penetration (Bharathi et al., 2024). Moreover, glycerol, being a cosolvent and permeation enhancer in nature could fluidize and disrupt the fungal cell wall and membrane packing, enhancing optimized VCZ delivery (Magdy et al., 2023). All the previous factors contributed to the superiority of VCZ OAG to VCZ suspension. It's also worth noting that there wasn't any observed antifungal activity (inhibition zone = zero mm) for blank OAG (supplementary fig. 1), confirming the attribution of superior antifungal activity of OAG solely to the enhanced penetration of VCZ caused by the collective of formulation components.

#### 3.6.2 Determination of the Minimum Inhibitory Concentration (MIC) and Minimum Fungicidal Concentration (MFC) and time to kill assay

The MIC of the OAG (1.95 μg/mL) was significantly lower than that of VCZ suspension (7.812 μg/mL) (t-test, *p* value<0.01, Fig. 4b). On the other hand, MIC of blank OAG was more than 500 μg/mL, confirming the absence of any antifungal activity of formulation components themselves.

Also, OAG showed a significantly lower MFC at 7.81 μg/mL (equivalent to four times the MIC value) than VCZ suspension whose MFC was 62.5 μg/mL (equivalent to eight times the MIC value).

Finally, OAG, at its MIC (1.95 μg/mL) killed the tested fungal strain after 10 h of incubation, while VCZ suspension, at its MIC (7.812 μg/mL) took longer time (24 h) to kill all the inoculated fungal culture, indicating a better antifungal activity of the OAG when compared to VCZ suspension.

According to the aforementioned values, our formulation showed comparable or lower MIC and MFC than reported VCZ formulations in the literature. A table summarizing the MIC and MFC values reported for standard VCZ is included (Supplementary File 2).

### 3.7 *In vivo* safety and performance studies of VCZ OAG

#### 3.7.1 Histopathological examination

**Fig. 5:**
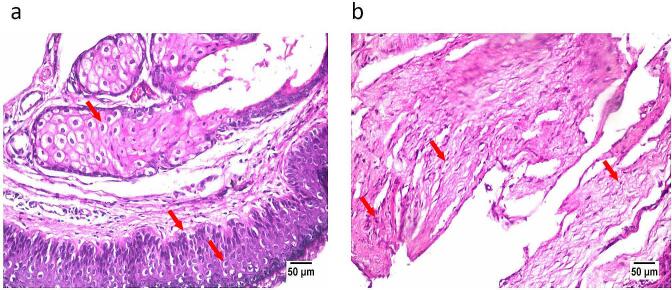
Microscopic photographs (16× magnification) showing normal histological structure of rabbit ear skin treated with (a) normal saline (negative control) and (b) optimal voriconazole augmented glycerosomes.

One key determinant of the validity of drug formulations for use is their safety. As demonstrated by Fig. 5, no observed ear skin histopathological changes were associated with the use of OAG, upon its comparison with the negative control. The test specimen maintained normal histological structure of the loose fibrous connective tissue. This concluded the safety of the used levels of all formulation ingredients and hence the safety of OAG for otic use.

#### 3.7.2 *In vivo* skin penetration

**Fig. 6:**
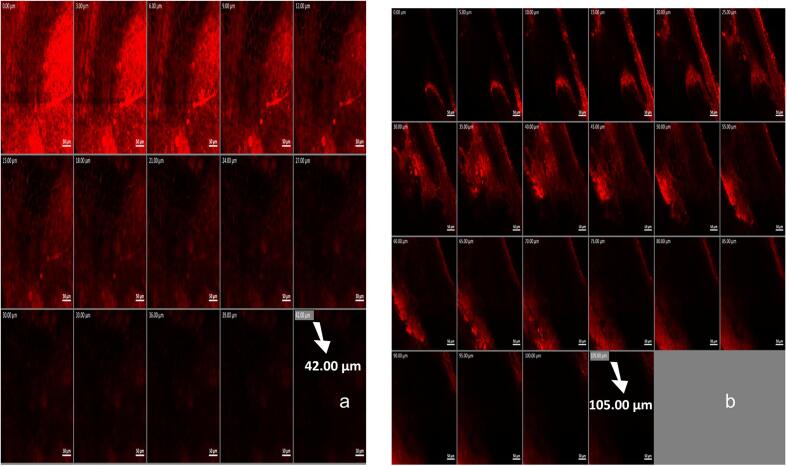
Confocal Laser Scanning Microscope micrographs showing different penetration depth of rhodamine B through rabbit ear skin from (a) aqueous solution and (b) optimal augmented glycerosomes.

As seen in Fig. 6, OAG could more deeply deliver RhB than the aqueous solution (105 vs 42 μm), confirming both its ability to deeply penetrate skin layers and the validity of the conducted *ex vivo* permeation study. The fact that all formulation ingredients are known to act as permeation enhancers can explain the obtained results, building toward a deeper skin penetration and delivery of VCZ, finally leading to successful therapeutic outcomes. (Guida et al., 2021).

## 4 Limitations and challenges

We have faced some limitations in our study. Although we reported the stability of our OAG for up to 3 months in the refrigerator, a longer stability study (6–12 months) is needed in the future to assess the stability of our formulations for practical and commercial applications. We, however, took an initiative to increase the stability by preparing a lyophilized OAG as it is known that dry formulations are generally more stable. We measured the main parameters (entrapment efficiency, vesicle size, zeta potential, and in *vitro* release profile) for the reconstituted formula and compared it to the original fresh formula. The results showed no significant difference in the parameters of the reconstituted formula and the fresh one, which implies that the lyophilization process did not negatively affect the formulation. The lyophilization protocol and the results are added as a supplementary (Supplementary File 1). We assume that large-scale production of our formulations might face some challenges, especially due to formulation complexity, need for advanced equipment, and stability concerns. We believe these challenges can be overcome with careful process optimization and rigorous quality control implementation.

Finally, while we believe that the experimental design for the animal study was informative, more animals would have given more statistical power to our results. Yet, we preferred to use a lower number of animals to stick with the 3R principles of animal research (reduce, reuse, and recycle).

## 5 Conclusion

Augmented voriconazole (VCZ) glycerosomes were successfully formulated via thin film hydration method according to a 2^3^ factorial design. The proposed optimal augmented glycerosomal formula (OAG) had the highest desirability value showing an optimal biphasic in *vitro* release profile and marked stability. Extensive *ex vivo*, microbiological, and *in vivo* studies concluded the safety of VCZ OAG and its superior performance compared to VCZ suspension. Thus, VCZ OAG could be regarded as a promising topical antiotomycotic formula for further investigations. Future directions would focus on formulation optimization to obtain a dry powder formula that can be reconstituted before administration (hence enhancing the stability and half-life of our formulations). It would also be interesting to investigate other antifungal drugs and administration routes.

## Funding

This research did not receive any specific grant from funding agencies in the public, commercial, or not-for-profit sectors.

## Ethics approval

The protocol for the *in vivo* study was approved by Research Ethics Committee of Faculty of Pharmacy, Cairo University (REC-FOPCU), Egypt (PI 3503), in compliance with ARRIVE guidelines and were carried out in accordance with the U.K. Animals (Scientific Procedures) Act, 1986 and associated guidelines, EU Directive 2010/63/EU for animal experiments.

## CRediT authorship contribution statement

**Sadek Ahmed:** Writing – review & editing, Resources, Investigation, Formal analysis, Conceptualization. **Heba Attia:** Writing – review & editing, Resources, Investigation, Formal analysis, Conceptualization. **Osama Saher:** Writing – review & editing, Resources, Investigation, Formal analysis, Conceptualization. **Abdurrahman M. Fahmy:** Writing – review & editing, Writing – original draft, Resources, Investigation, Formal analysis, Conceptualization.

## Declaration of competing interest

The authors declare that they have no known competing financial interests or personal relationships that could have appeared to influence the work reported in this paper.

## Data Availability

The datasets generated during and/or analyzed during the current study are available from the corresponding author on reasonable request.

## Appendix A Supplementary data

Supplementary material 1: Supplementary Fig. 1 Antifungal activity determination by Kirby–Bauer disk diffusion technique. A: The inhibition zone caused by 5 μg VCZ loaded on a sterile filter paper disk. B: The inhibition zone caused by 5 μg VCZ OAG loaded on a sterile filter paper disk. C. The inhibition zone caused by 5 μg blank OAG loaded on a sterile filter paper disk (zero mm). Right panel shows the back of the same petri dish in the left panel. Supplementary Fig. 2 Additional Confocal Laser Scanning Microscope micrographs showing different penetration depth of rhodamine B through rabbit ear skin from (a) aqueous solution and (b) optimal augmented glycerosomes Image 1

Supplementary Table 2: MIC and MFC values reported in the literature for standard VCZ Image 2
