# Supplementary material for: Augmented glycerosomes as a promising approach against fungal ear infection: Optimization and microbiological, ex vivo and in vivo assessments
Source: Int J Pharm X. 2024 Oct 22;8:100295. doi: 10.1016/j.ijpx.2024.100295 (PMC11543555; doi:10.1016/j.ijpx.2024.100295)
Supplement: Supplementary file 1 — Supplementary material 1: Supplementary Fig. 1 Antifungal activity determination by Kirby–Bauer disk diffusion technique. A: The inhibition zone caused by 5 μg VCZ loaded on a sterile filter paper disk. B: The inhibition zone caused by 5 μg VCZ OAG loaded on a sterile filter paper disk. C. The inhibition zone caused by 5 μg blank OAG loaded on a sterile filter paper disk (zero mm). Right panel shows the back of the same petri dish in the left panel. Supplementary Fig. 2 Additional Confocal Laser Scanning Microscope micrographs showing different penetration depth of rhodamine B through rabbit ear skin from (a) aqueous solution and (b) optimal augmented glycerosomes [file mmc1.docx]

**Methodology**

**Preparation of freeze-dried glycerosomes**

Formulations were prepared using the same thin film hydration method with inclusion of 5 % *w/v* mannitol as cryoprotectant in the aqueous phase prior to being stored in a freezer at −22 °C for 24 h.

The frozen solutions were then placed in a lyophilizer (Novalyphe-NL 500; Savant Instruments Corp., Holbrook, NY, USA) for 24 h. The condenser temperature was—45 °C and under a vacuum of 7 × 10^−2^ mBAR.

Evaluation of the parameters after reconstitution was done using the same methodologies described in the main manuscript.

**Results**

Supplementary Table 1: Effect of Lyophilization on Voriconazole Optimal Augmented Glycerosomes

|  | **Fresh** | **Reconstituted** | **P-value** |
| --- | --- | --- | --- |
| EE | 84.3±2.0 | 80.58±2.97 | **0.278** |
| VS (nm) | 191±1.1 | 200.9±3.54 | **0.064** |
| ZP (mV) | -38.8±1.8 | -33.3±1.7 | **0.090** |


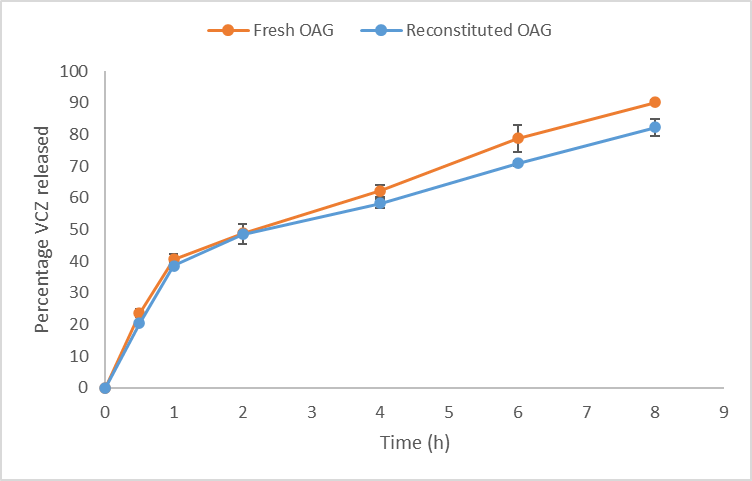

$$f_{2}=64.23$$

**Supplementary Figure 1.** *In vitro* release profiles of fresh liquidoptimal voriconazole augmented glycerosomes, compared to the reconstituted lyophilized analogue
