# Supplementary material for: Augmented glycerosomes as a promising approach against fungal ear infection: Optimization and microbiological, ex vivo and in vivo assessments
Source: Int J Pharm X. 2024 Oct 22;8:100295. doi: 10.1016/j.ijpx.2024.100295 (PMC11543555; doi:10.1016/j.ijpx.2024.100295)
Supplement: Supplementary file 2 — Supplementary Table 2: MIC and MFC values reported in the literature for standard VCZ [file mmc2.docx]

| Strain | MIC (μg/mL) | MFC (μg/mL) | Reference |
| --- | --- | --- | --- |
| *Aspergillus calidoustus* (FMR  13556) | 8 | - | (1) |
| *Aspergillus calidoustus* (FMR  13557) | 16 | - | (1) |
| *Aspergillus fumigatus* (FMR 10522) | 1 | - | (1) |
| *Aspergillus fumigatus* clinical isolates | 0.5 | 8 | (2) |
| *Aspergillus flavus* ATCC 204304 | 0.5 | - | (1) |
| *Aspergillus flavus* clinical isolates | 0.0313-1 | - | (3) |
| *Aspergillus flavus* clinical isolates | - | 0.5-2 | (4) |
| *Aspergillus flavus* clinical isolates | 60-500 | - | (5) |
| *Aspergillus flavus* clinical isolates | 0.5 | 4 | (2) |
| *Aspergillus fumigatus* clinical isolates | 0.023–3 | - | (6) |
| *Aspergillus fumigatus* clinical isolates | 0.0313-1 | - | (3) |
| *Aspergillus fumigatus* clinical isolates | 60-130 | - | (5) |
| *Aspergillus niger* clinical isolates | 0.5 | - | (7) |
| *Aspergillus niger* clinical isolates | 0.032–5 | - | (6) |
| *Aspergillus niger* clinical isolates | 0.094–0.75 | - | (6) |
| *Aspergillus niger* clinical isolates | 50-120 | - | (5) |
| *Aspergillus niger* clinical isolates | - | 1-4 | (4) |
| *Aspergillus niger* clinical isolates | 0.0313-1 | - | (3) |
| *Aspergillus terreus* clinical isolates | 0.125–0.5 | - | (6) |
| *Aspergillus terreus* clinical isolates | 1 | 8 | (2) |
| *Aspergillus terreus* clinical isolates | - | 0.5-1 | (4) |

Supplementary Table 1: MIC and MFC values reported in the literature for standard VCZ

**References**

1. Gallego-Arranz T, Perez-Cantero A, Torrado-Salmeron C, Guarnizo-Herrero V, Capilla J, Torrado-Duran S. Improvement of the pharmacokinetic/pharmacodynamic relationship in the treatment of invasive aspergillosis with voriconazole. Reduced drug toxicity through novel rapid release formulations. Colloids Surf B Biointerfaces. 2020;193:111119.

2. Meletiadis J, Antachopoulos C, Stergiopoulou T, Pournaras S, Roilides E, Walsh TJ. Differential fungicidal activities of amphotericin B and voriconazole against *Aspergillus* species determined by microbroth methodology. Antimicrob Agents Chemother. 2007;51(9):3329-37.

3. Misra R, Malik A, Singhal S. Comparison of the activities of amphotericin B, itraconazole, and voriconazole against clinical and environmental isolates of *Aspergillus* species. Indian J Pathol Microbiol. 2011;54(1):112-6.

4. Lass-Florl C, Nagl M, Speth C, Ulmer H, Dierich MP, Wurzner R. Studies of *in vitro* activities of voriconazole and itraconazole against *Aspergillus* hyphae using viability staining. Antimicrob Agents Chemother. 2001;45(1):124-8.

5. Gheith S, Saghrouni F, Bannour W, Ben Youssef Y, Khelif A, Normand AC, et al. *In vitro* susceptibility to amphotericin B, itraconazole, voriconazole, posaconazole and caspofungin of *Aspergillus* spp. isolated from patients with haematological malignancies in Tunisia. Springerplus. 2014;3:19.

6. Mallie M, Bastide JM, Blancard A, Bonnin A, Bretagne S, Cambon M, et al. *In vitro* susceptibility testing of *Candida* and *Aspergillus* spp. to voriconazole and other antifungal agents using Etest: results of a French multicentre study. Int J Antimicrob Agents. 2005;25(4):321-8.

7. Kaya AD, Kiraz N. *In vitro* susceptibilities of *Aspergillus* spp. causing otomycosis to amphotericin B, voriconazole and itraconazole. Mycoses. 2007;50(6):447-50.
